# In Vivo Evidence of Reduced Integrity of the Gray–White Matter Boundary in Autism Spectrum Disorder

**DOI:** 10.1093/cercor/bhw404

**Published:** 2017-01-05

**Authors:** Derek Sayre Andrews, Thomas A. Avino, Maria Gudbrandsen, Eileen Daly, Andre Marquand, Clodagh M. Murphy, Meng-Chuan Lai, Michael V. Lombardo, Amber N.V. Ruigrok, Steven C. Williams, Edward T. Bullmore, The MRC AIMS Consortium, John Suckling, Simon Baron-Cohen, Michael C. Craig, Declan G.M. Murphy, Christine Ecker

**Affiliations:** 1 Department of Forensic and Neurodevelopmental Sciences, Sackler Institute for Translational Neurodevelopment, Institute of Psychiatry, Psychology and Neuroscience, King's College London, London SE5 8AF, UK; 2 Department of Psychiatry and Behavioral Sciences, M.I.N.D. Institute, University of California Davis, Sacramento, CA, USA; 3 Donders Institute for Brain, Cognition and Behaviour, Radboud University, Nijmegen, The Netherlands; 4 Centre for Neuroimaging Sciences, Institute of Psychiatry, Psychology and Neuroscience, King's College, London, UK; 5 National Autism Unit, Bethlem Royal Hospital, South London and Maudsley NHS Foundation Trust, London, UK; 6 Autism Research Centre, Department of Psychiatry, University of Cambridge, Cambridge, UK; 7 Child and Youth Mental Health Collaborative at the Centre for Addiction and Mental Health and The Hospital for Sick Children, Department of Psychiatry, University of Toronto, Toronto, Canada; 8 Department of Psychiatry, National Taiwan University Hospital and College of Medicine, Taipei, Taiwan; 9 Department of Psychology & Center for Applied Neuroscience, University of Cyprus, Nicosia, Cyprus; 10 Brain Mapping Unit, Department of Psychiatry, University of Cambridge, Cambridge, UK; 11 The Medical Research Council Autism Imaging Multicentre Study Consortium (MRC AIMS Consortium) is a UK collaboration between the Institute of Psychiatry, Psychology and Neuroscience at King's College, London, the Autism Research Centre, University of Cambridge, and the Autism Research Group, University of Oxford. The Consortium members in alphabetical order are as follows: Anthony J. Bailey (Oxford), Simon Baron-Cohen (Cambridge), Patrick F. Bolton (IoP), Edward T. Bullmore (Cambridge), Sarah Carrington (Oxford), Marco Catani (IoPPN), Bhismadev Chakrabarti (Cambridge), Michael C. Craig (IoPPN), Eileen M. Daly (IoPPN), Sean C.L. Deoni (IoPPN), Christine Ecker (IoPPN), Francesca Happé (IoPPN), Julian Henty (Cambridge), Peter Jezzard (Oxford), Patrick Johnston (IoPPN), Derek K. Jones (IoPPN), Meng-Chuan Lai (Cambridge), Michael V. Lombardo (Cambridge), Anya Madden (IoPPN), Diane Mullins (IoPPN), Clodagh M. Murphy (IoPPN), Declan G.M. Murphy (IoPPN), Greg Pasco (Cambridge), Amber N.V. Ruigrok (Cambridge), Susan A. Sadek (Cambridge), Debbie Spain (IoPPN), Rose Stewart (Oxford), John Suckling (Cambridge), Sally J. Wheelwright (Cambridge), Steven C. Williams (IoPPN), and C. Ellie Wilson (IoPPN); 12 Department of Child and Adolescent Psychiatry, Psychosomatics and Psychotherapy, Universitätsklinikum Frankfurt am Main, Goethe-University Frankfurt am Main, Frankfurt, Germany

**Keywords:** ASD, FreeSurfer, imaging, lamination, MRI

## Abstract

Atypical cortical organization and reduced integrity of the gray–white matter boundary have been reported by postmortem studies in individuals with autism spectrum disorder (ASD). However, there are no in vivo studies that examine these particular features of cortical organization in ASD. Hence, we used structural magnetic resonance imaging to examine differences in tissue contrast between gray and white matter in 98 adults with ASD and 98 typically developing controls, to test the hypothesis that individuals with ASD have significantly reduced tissue contrast. More specifically, we examined contrast as a percentage between gray and white matter tissue signal intensities (GWPC) sampled at the gray–white matter boundary, and across different cortical layers. We found that individuals with ASD had significantly reduced GWPC in several clusters throughout the cortex (cluster, *P* < 0.05). As expected, these reductions were greatest when tissue intensities were sampled close to gray–white matter interface, which indicates a less distinct gray–white matter boundary in ASD. Our in vivo findings of reduced GWPC in ASD are therefore consistent with prior postmortem findings of a less well-defined gray–white matter boundary in ASD. Taken together, these results indicate that GWPC might be utilized as an in vivo proxy measure of atypical cortical microstructural organization in future studies.

## Introduction

Autism spectrum disorder (ASD) is a lifelong neurodevelopmental condition characterized by impaired social communication, deficits in social reciprocity, and repetitive and stereotypic behaviors and interests (Wing 1997). These core symptoms typically manifest from early childhood, and are accompanied by developmental differences in brain anatomy and connectivity (for review, see Amaral et al. 2008; Ecker et al. 2015; Lange et al. 2015). For example, prior studies of ASD reported atypical measures of cortical anatomy such as folding, thickness, and surface area (Nordahl et al. 2007; Hyde et al. 2010; Schaer et al. 2013; Ecker et al. 2013a) as well as intra-cortical connectivity (Ecker et al. 2013b). However, the causes of these cortical abnormalities in people with ASD are unknown.

There is some evidence to suggest that the cortical differences accompanying ASD may result from atypical neuronal proliferation, migration, and maturation (Pinto et al. 2014). For example, some genetic variants associated with ASD encode for genes that regulate these neurodevelopmental processes (Huguet et al. 2013). It has been suggested that these variations may explain postmortem findings such as irregular cortical lamination, the presence of super-numerous neurons in some layers of the cortex, and poor differentiation of the gray–white matter boundary (for review, see Casanova 2014). For example, histological samples from the superior temporal gyrus (approximate Brodmann area [BA] 21), dorsolateral frontal lobe (BA9) and dorsal parietal lobe (BA7) have shown the gray–white matter boundary to be less distinct in ASD as compared with typically developing (TD) controls (Avino and Hutsler 2010). Thus, there is increasing postmortem evidence for abnormal cell patterning within the gray–white matter boundary in ASD. However, to date no study has investigated differences in the integrity of the gray–white matter boundary in ASD in vivo across the whole brain.

Current in vivo neuroimaging methods for investigating cortical abnormalities in ASD focus on morphometric features such as cortical thickness (CT), that is, the closest distance from the gray–white matter boundary to the gray-cerebral spinal fluid (CSF) boundary (Fischl and Dale 2000). Differences in CT have been reported in children, adolescents, and adults with ASD, and include regional increases and decreases that may mediate some of the behavioral deficits typically observed in the disorder (Hardan et al. 2006; Hyde et al. 2010; Ecker et al. 2013b). However, measures of CT rely on the accurate delineation of gray and white matter and therefore may be confounded by intrinsic histological abnormalities at the gray–white matter boundary in ASD (Avino and Hutsler 2010).

Hence, we investigated between-group differences related to cortical lamination in both adult males and females with ASD, and matched TD controls, using a whole brain quantitative approach that estimated integrity of the gray–white matter boundary. Namely we examined the percent contrast of gray-to-white matter signal intensities (GWPC), sampled across different cortical layers in a continuous fashion. Here, the GWPC calculation we employed in the current manuscript is comparable to the gray–white contrast ratio as originally reported by Salat et al. (2009). We hypothesized the gray–white matter boundary to be less defined and therefore GWPC to differ significantly in individuals with ASD.

## Materials and Methods

### Participants

Overall, 98 right-handed adults with ASD (49 males and 49 females) and 98 age, sex, and IQ matched TD controls (51 males and 47 females) aged 18–42 years were recruited by advertisement and assessed at the Institute of Psychiatry, Psychology and Neuroscience (IoPPN), London, and the Autism Research Centre, Cambridge. Approximately equal ratios of cases to controls, and males to females, were recruited within sites (Table 1). Exclusion criteria included a history of major psychiatric disorder (e.g. psychosis), head injury, genetic disorder associated with autism (e.g. fragile-X syndrome, tuberous sclerosis), or any other medical condition affecting brain function (e.g. epilepsy), or any participants taking antipsychotic medication, mood stabilizers or benzodiazepines.

**Table 1:** Participant demographics

	ASD (*n* = 98 [49♂, 49♀])	Control (*n* = 98 [51♂, 47♀])
London	*n* = 45 (24♂, 21♀)	*n* = 44 (25♂, 19♀)
Cambridge	*n* = 53 (25♂, 28♀)	*n* = 54 (26♂, 28♀)
Age, years	26 ± 7 (18–48)	27 ± 6 (18–52)
Full-scale IQ, WASI	113 ± 12 (84–136)	116 ± 9 (93–137)
ADI-R social^a^	17 ± 5 (10–28)	*
ADI-R communication^a^	13 ± 4 (2–24)	*
ADI-R repetitive behavior^a^	5 ± 2 (1–10)	*
ADOS social + communication^b^	9 ± 5 (0–21)	*

Data expressed as mean ± standard deviation (range). There were no significant between-group differences in age or IQ, *P* < 0.05 (2-tailed). All participants were diagnosed using ICD-10 criteria.

^a^The Autism Diagnostic Interview Revised (ADI-R) was used to confirm ASD diagnosis. ADI-R scores were unavailable for 4 participants. Each of these cases reached.

^b^The Autism Diagnostic Observation Schedule (ADOS) cut-offs for “autism spectrum”, for all other participants the ADOS was not used as diagnostic criteria.

*TD controls did not undergo ADI-R or ADOS assessments.

ASD diagnosis was made by a consultant psychiatrist using ICD-10 research diagnostic criteria and confirmed using the ADI-R (Lord et al. 1994). ADI-Rs were completed for 94 individuals with ASD (49 males and 45 females). Ninety-three (49 males and 44 females) reached algorithm cut-offs for autism in all domains of the ADI-R (social, communication, and restricted/stereotyped), although failure to reach cut-off in one domain by one point was permitted. The ADI-R rather than ADOS (Lord et al. 2000) was employed as inclusion criteria to ensure that all participants with ASD met the criteria for childhood autism. We were unable to complete ADI-Rs for 4 females with ASD as their parents/caregivers were not available. However, all 4 reached algorithm cut-offs for “autism spectrum” on the ADOS (communication, social) diagnostic algorithm. In all other participants, ADOS scores were used to measure current symptoms and not as inclusion criterion. One ASD female scored one point below cut-off for autism on the communication and repetitive behavior domains of the ADI-R but met ICD-10 criteria for ASD and scored above cut-off for “autism” on the ADOS. Overall intellectual ability was assessed using the Wechsler Abbreviated Scale of Intelligence (WASI; Wechsler 1999). All participants had a full-scale IQ greater than 80 and gave informed written consent in accordance with ethics approval by the National Research Ethics Committee, Suffolk, UK.

### Structural MRI Data Acquisition

Scanning was performed at the IoPPN, London, and Addenbrooke's Hospital, Cambridge, using a 3-T GE Signa System (General-Electric). A specialized acquisition protocol using quantitative T1-mapping was used to ensure standardization of structural magnetic resonance imaging (MRI) scans across scanner platforms. This protocol has previously been validated and extensively described elsewhere (Deoni et al. 2008; Ecker et al. 2012), resulting in high-resolution structural T1-weighted inversion-recovery images, with 1 × 1 × 1 mm resolution, a 256 × 256 × 176 matrix, TR = 1800 ms, TI = 50 ms, FA = 20″, and FOV = 5 cm.

### Cortical Reconstruction Using FreeSurfer

Previous histological studies have largely relied upon manual identification to define the boundary between gray and white matter. For example, Avino and Hutsler (2010) used a sigmoid function to quantify the distinctiveness of the transition between gray and white matter in Nissl-stained histological images. In the current study, however, we employed an automated analytical pipeline using FreeSurfer v5.3.0 software (http://surfer.nmr.mgh.harvard.edu/) to identify the gray–white matter boundary by deriving models of the cortical surface for each T1-weighted image. These well-validated and fully automated procedures have been detailed elsewhere (Dale et al. 1999; Fischl et al. 1999; Fischl and Dale 2000; Ségonne et al. 2004; Jovicich et al. 2006). In brief, a single-filled white-matter volume was generated for each hemisphere after intensity normalization, extra-cerebral tissue was cropped, and image segmentation performed using a connected components algorithm. A triangular tessellated surface was then generated for each white-matter volume. Deformation of this tessellated white matter surface resulted in a cortical mesh for the surfaces that define the boundary between gray and white matter (i.e. white matter surface), and gray matter and CSF (i.e. pial surface). This surface deformation is the result of the minimization of an energy functional that utilizes intensity gradients in order to place these surfaces where the greatest shift in intensity defines the transition between tissue classes (Dale et al. 1999, Supplementary Material). The use of intensity gradients across tissue classes assures that boundary placement is not reliant solely on absolute signal intensity and allows for subvoxel resolution in the placement of these boundary surfaces (Dale and Sereno 1993; Dale et al. 1999; Fischl and Dale 2000). These automated methods have previously been validated against histological analyses and have shown a high degree of accuracy in placing the gray–white matter boundary (Rosas et al. 2002). The resulting surface models were visually inspected for reconstruction errors. Participant's surface reconstructions with visible inaccuracies were excluded and are not described in this study. Dropout rates due to surface reconstruction errors were equal between groups and represented <10% of the total sample.

### Gray-to-White Matter Percent Contrast (GWPC) and Gray-Matter Signal Intensity Measures

**Figure 1.**
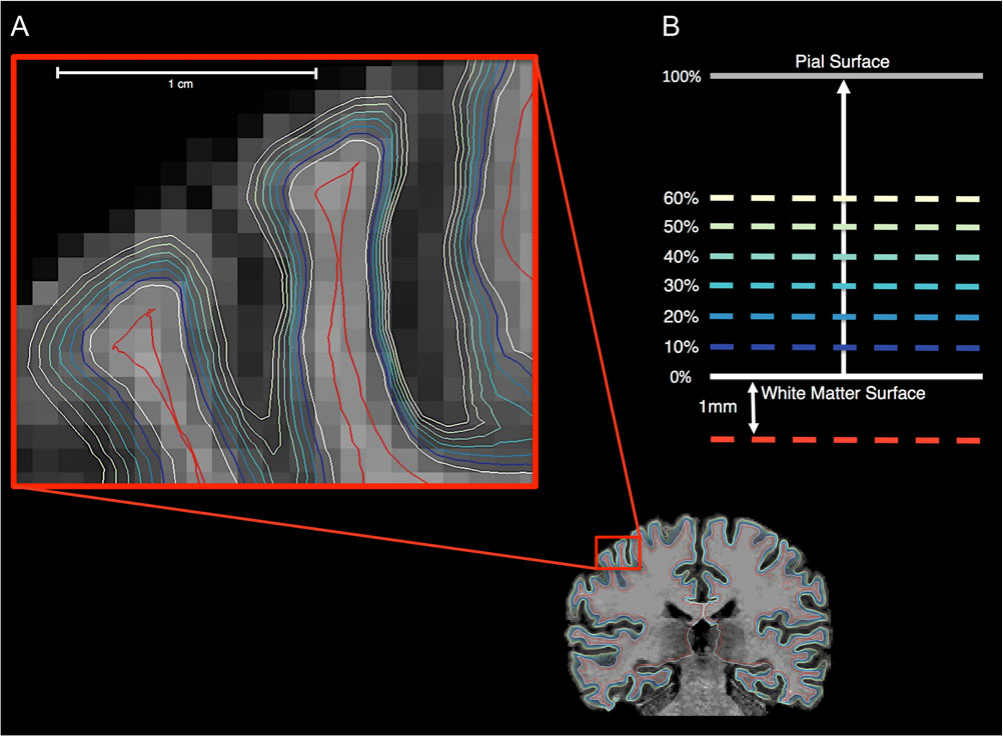
Gray and white matter signal intensity sampling procedure. (*A*) Gray and white matter signal intensity sampling points are shown for one 2D coronal slice. (*B*) WMIs (red line) were sampled at an absolute distance of 1 mm subjacent to the white matter surface (i.e. gray–white matter boundary). GMI signals (blue to yellow lines) were measured at projection fractions representing a percentage of the total orthogonal distance from the white matter surface to the outer pial surface starting at the white matter surface up to 60% into the cortical sheet at 10% intervals.

Gray-matter tissue intensities (GMI)  were sampled continuously across different cortical layers from the gray–white matter boundary (i.e. white matter surface) to the pial surface. These signal intensities were sampled at different percentile fractions of the total orthogonal distance projected from the white matter to pial surfaces (i.e. projection fractions). Starting at the white matter surface, sampling continued at projection fraction intervals of 10% up to 60% of the distance from the white matter to the pial surface, thus yielding a set of 6 GMI measures (i.e. from 10% to 60%; Fig. 1). The outer 40% (i.e. 70–100%) of the cortical sheet was not sampled in order to assure that sampling was performed within the cortical gray matter, and not confounded by voxels composed of CSF. White matter signal intensity (WMI) was measured at 1.0 mm into the white matter from the white matter surface (Fig. 1). Previously reported measures of tissue contrast have used a ratio calculation (i.e. GMI/WMI; Salat et al. 2009), where larger values indicate a reduced contrast. Here, however, we utilized the formula provided by FreeSurfer to calculate tissue contrast as the percentage of GMI at projection fraction (*i*) to WMI at each cerebral vertex (*j*),

$$\frac{GWPC_{ij}=100\times (WMI_{i,1.0mm}-GMI_{i,j})}{0.5\times (WMI_{i,1.0mm}+GMI_{i,j})}.$$

Thus, by definition, a decrease in GWPC is commensurate with a decrease in contrast between the GMI measured at projection fraction *i*, and the WMI measured at 1.0 mm subjacent to the white matter surface. We also examined the tissue contrast when sampling GMI at the gray–white matter boundary (i.e. at the white matter surface, projection fraction = 0%). The resulting GWPC, GMI, and WMI measures were subsequently smoothed using a 10-mm full-width at half-maximum (FWHM) surface-based Gaussian kernel prior to statistical analyses. We also examine between-group comparisons using a 5-mm FWHM smoothing kernel, which are shown in Supplementary Figure 3 and Table 3.

### Statistical Analyses

Vertex-wise statistical analysis of GWPC, GMI, and WMI measures (*Y*) were estimated by the regression of a general linear model (GLM) with 1) diagnostic group, sex, and acquisition site as categorical fixed-effects factors, 2) a group by sex interaction term, and 3) age and full-scale IQ as continuous covariates:

$$\begin{array}{c} Y_{i}=\beta_{0}+\beta_{1}Group+\beta_{2}Sex+\beta_{3}\left[Group\,x\,Sex\right]+\beta_{4}Site+\beta_{5}Age\\ +\beta_{6}FSIQ+\varepsilon_{i}, \end{array}$$

where *ε*_*i*_ is the residual error at vertex *i*. Between-group differences were estimated from the corresponding coefficient *β*_1_, normalized by the corresponding standard error. Our model was selected a priori in order to be comparable to previously published research findings based on our sample (Ecker et al. 2013b). Corrections for multiple comparisons across the whole brain were performed using “random field theory” (RFT)-based cluster analysis for non-isotropic images using a cluster-based significance threshold of *P* < 0.05 (2-tailed; Worsley et al. 1999). Initially, we investigated between-group differences in GWPC at different gray-matter projection fractions. Subsequently, we also investigated between-group differences in gray and white matter tissue intensities, which allowed us to determine whether the between-group differences in GWPC were driven by differences within the cortical gray or white matter. Last, between-group differences in CT were examined using the same GLM as described above in order to determine how differences in GWPC might affect variability in CT in ASD.

## Results

### Participant Demographics and Global Brain Measures

There were no significant differences between individuals (males and females) with ASD and TD controls in age (*t*(194) = −0.53,*P*= 0.598), full-scale IQ (*t*(194) = −1.72,*P*= 0.086), or total GM volume (*t*(194) = −0.20,*P*= 0.839). There were also no significant differences between males and females in age (*t*(194) = −0.93,*P*= 0.356) or full-scale IQ (*t*(194) = −1.87,*P*= 0.063). As expected, total gray matter volume in males was significantly larger than in females (*t*(194) = 9.11, *P* < 0.001). However, there were no significant differences in any of these measures between males with ASD and male controls, or females with ASD and female controls (*P* < 0.05, 2-tailed).

### Between-Group Difference in GWPC Across the Cortex

**Figure 2.**
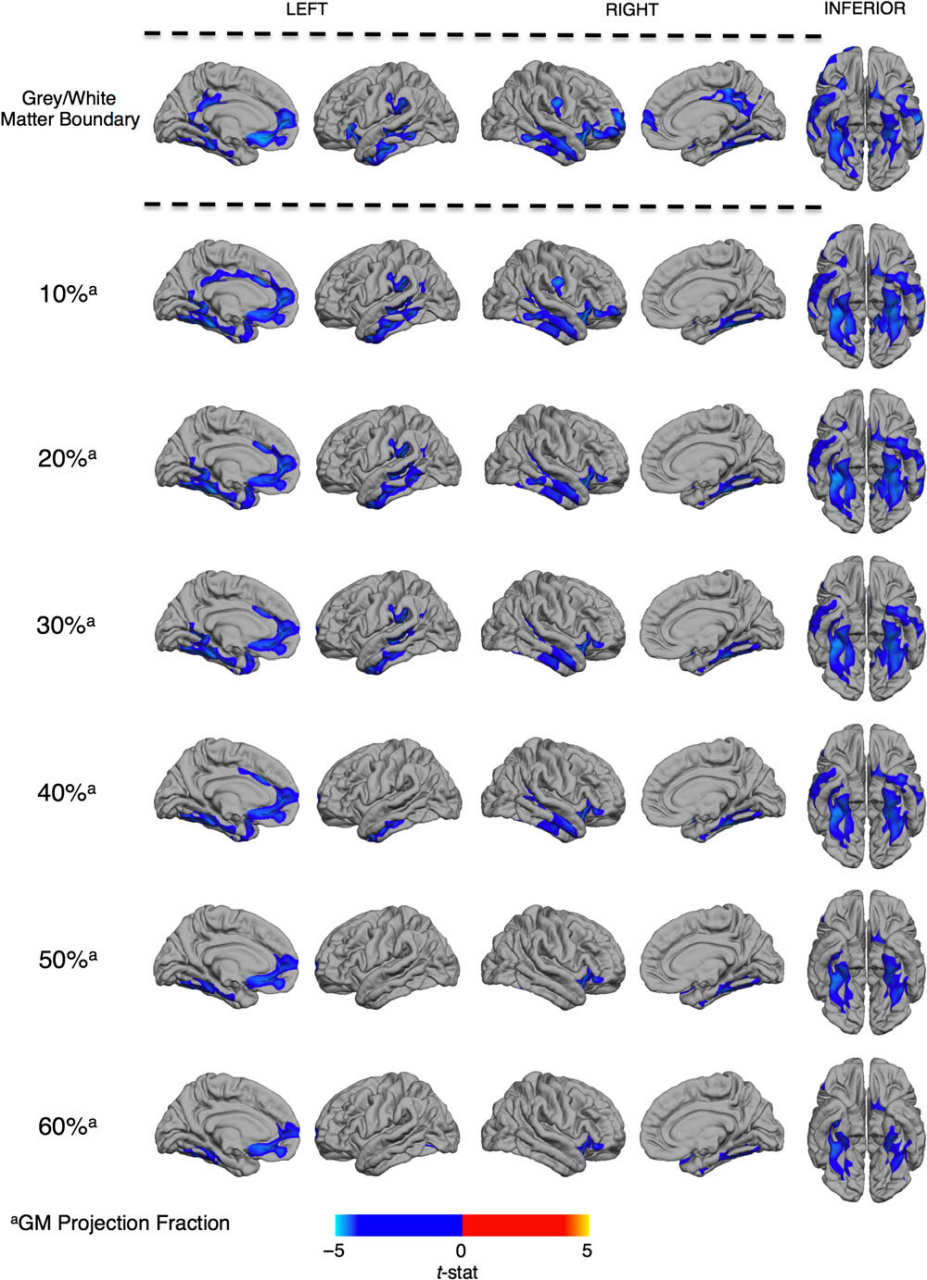
Regions of decreased gray-to-white matter signal intensity percent contrast (GWPC) in ASD. Individuals with ASD showed significantly decreased GWPC (RFT, *P* < 0.5), indicating less definition between gray and white matter, in several regions highlighted in blue including 1) the posterior-cingulate cortex, 2) fronto-temporal and fronto-parietal regions, as well as 3) the bilateral fusiform and entorhinal cortex. The spatial and statistical extent of these differences was greatest when tissue intensities were sampled at the gray–white matter boundary and decreased along with increasing projection fractions (superscript a) into the cortical sheet. See Table 2 for statistical details.

We initially examined vertex-wise between-group differences in GWPC at different projection fractions into the cortical sheet. At all sampling depths, we found that individuals with ASD had a significantly decreased GWPC in several clusters across the cortex, which is consistent with a reduced tissue contrast between gray and white matter (Fig. 2). In accordance with our hypothesis, the reductions in GWPC were most extensive when GMI was sampled at gray–white matter boundary (i.e. the white matter surface, projection fraction = 0%), and gradually decreased in both statistical effect and spatial extent with increasing projection fractions into cortex and away from the gray–white matter boundary. Regions where ASD individuals had reduced GWPC as compared with TD controls included the 1) bilateral posterior-cingulate (BA 23/30), medial frontal (BA10) fusiform/entorhinal (BA 34/37) and the inferior and superior temporal cortices (BA20/21/22); 2) left orbitofrontal cortex (BA11/25) and temporo-parietal junction (BA39/40); and (3) right dorsolateral prefrontal cortex (BA11/45). Statistical details for all clusters are listed in Table 2. There were no brain regions where individuals with ASD had a significantly increased GWPC relative to controls. The pattern of reduced GWPC among individuals with ASD remained significant when total brain volume or mean CT were included as covariates. Furthermore, there was minimal spatial overlap between the pattern of differences in GWPC and CT (see Supplementary Fig. 1 and Table 1).

**Table 2:** Clusters of significant reductions in gray white matter percent contrast and increases in gray matter intensity in ASD

Measure	Cluster	Region labels	Hemisphere	BA (*t*max)	Vertices	Talairach			*t*_max_	*p*_cluster_
						*x*	*y*	*z*		
GWPC	1	Superior temporal gyrus, insula, lateral orbital frontal cortex, pars orbitalis, pars triangularis, postcentral gyrus, precentral gyrus, rostral middle frontal gyrus, superior frontal gyrus	L	21	10 204	47	−4	−14	−3.95	4.38 × 10^–6^
	2	Posterior-cingulate cortex, isthmus-cingulate cortex, lingual gyrus, precuneus cortex	R	31	5760	7	−30	39	−3.77	2.05 × 10^–5^
	3	Middle temporal gyrus, banks superior temporal sulcus, inferior temporal gyrus, superior temporal gyrus	R	21	4994	54	−11	−18	−3.87	4.48 × 10^–5^
	4	Middle temporal gyrus, banks superior temporal sulcus, inferior temporal gyrus, superior temporal gyrus	L	21	4837	−53	−20	−3	−3.59	1.46 × 10^–5^
	5	Insula, lateral orbital frontal cortex, pars opercularis, postcentral gyrus, precentral gyrus	L	13	4168	−27	24	−1	−3.64	1.68 × 10^–3^
	6	Parahippocampal gyrus, fusiform gyrus, lingual gyrus	R	19	4053	25	−53	−2	−3.34	7.63 × 10^–4^
	7	Medial orbital frontal cortex, rostral anterior cingulate cortex, superior frontal gyrus	L	11	3520	−8	25	−14	−4.13	2.14 × 10^–3^
	8	Fusiform gyrus, lingual gyrus, parahippocampal gyrus	L	37	3443	−36	−42	−8	−3.26	6.62 × 10^–3^
	9	Posterior-cingulate cortex, isthmus-cingulate cortex, lingual gyrus, precuneus cortex	L	23	3432	−8	−56	16	−3.33	3.92 × 10^–3^
	10	Supramarginal gyrus	L	40	2466	−56	−32	27	−3.15	3.26 × 10^–3^
GMI	1	Superior temporal gyrus, banks superior temporal sulcus, fusiform gyrus, inferior parietal cortex, inferior temporal gyrus, insula, isthmus-cingulate cortex, lateral orbital frontal cortex, lingual gyrus, middle temporal gyrus, parahippocampal gyrus, pars triangularis, supramarginal gyrus, temporal pole	R	38	17 938	35	5	−10	4.02	1.69 × 10^–6^
	2	Superior temporal gyrus, banks superior temporal sulcus, inferior parietal cortex, inferior temporal gyrus, middle temporal gyrus	L	21	10 279	−51	−26	−2	3.31	2.86 × 10^–5^
	3	Fusiform gyrus, inferior temporal gyrus, isthmus-cingulate cortex, lingual gyrus, precuneus cortex	L	37	6295	−44	−40	−14	3.27	2.70 × 10^–3^

Notes: Clusters of significant reductions in GWPC and increases in gray matter intensity (GMI) in ASD: BA, left (L), right (R), ‘Vertices’ indicates the number of vertices within the cluster, *t*_max_ represents the maximum *t*-statistic within the cluster located at the *x y z* Talairach coordinates listed, *p*_cluster_ is the cluster corrected *P* value.

### Between-Group Differences in Gray and White Matter Tissue Intensities

**Figure 3.**
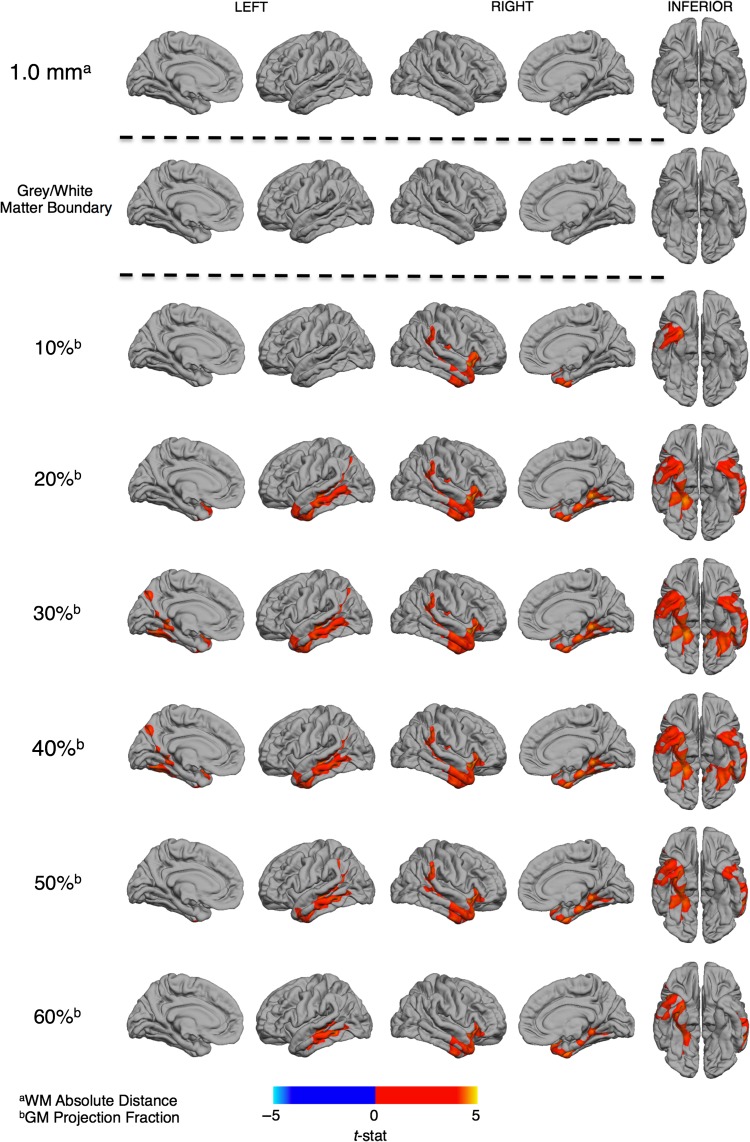
Regional differences in gray (GMI) and white matter (WMI) signal intensities in ASD. Individuals with ASD showed no significant differences in WMI (RFT, *P* < 0.5) measured at 1 mm subjacent to the gray–white matter boundary (superscript a) nor tissue intensities measured at the boundary. Significantly increased GMI (RFT, *P* < 0.5) was observed across all projection fractions (superscript b) within the cortical sheet in ASD participants. The statistical and spatial extent of these increases in GMI were most evident at the 30% projection fraction and incorporated 1) the bilateral anterior temporal lobes and the left middle temporal gyrus, 2) the right temporo-parietal junction, and 3) the bilateral fusiform and entorhinal cortex. See Table 2 for statistical details.

To identify whether the observed differences in GWPC were driven by differences in gray or white matter, or a combination of both, we subsequently examined between-group differences in both GMI and WMI. Individuals with ASD had significantly increased GMI across all 6 different GMI sampling depths relative to controls in regions where we also observed decreases in GWPC (Fig. 3). These included 1) the bilateral anterior temporal lobes (BA38/30) and the left middle temporal gyrus (BA21), 2) the right temporo-parietal junction (BA39/40), and 3) the bilateral fusiform and entorhinal cortex (BA36). Statistical details for these clusters are listed in Table 1. We did not observe any significant between-group differences in GMI at the gray–white matter boundary (i.e. the white matter surface), or in WMI at 1.0 mm within the white matter (Fig. 3). There were no brain regions where individuals with ASD had significantly decreased GMI relative to controls. Hence, GWPC reductions in ASD were driven predominantly by increased (i.e. brighter) tissue intensities within the cortical gray matter.

### Main Effects of Sex and Group by Sex Interactions

Last, we investigated whether biological sex significantly modulates differences in GWPC in ASD by examining group-by-sex interactions. Overall, regardless of diagnosis, males had a significantly greater GWPC than females (Supplementary Fig. 2). This occurred across all sampling depths, and was predominantly in fronto-parietal regions of the left hemisphere, and in bilateral inferior temporal regions (see Supplementary Table 2 for statistical details of these clusters). However, there were no brain regions where we observed significant group-by-sex interactions for GWPC. Thus, while males tended to have a significant increase in contrast between gray and white matter tissue intensities the reductions in GWPC that we observed in the brain in individuals with ASD were not explained by biological sex.

## Discussion

Our aim was to determine if previous postmortem reports of poor definition of the gray–white matter boundary in ASD could be detected using a whole brain in vivo MRI approach. As hypothesized, we determined that individuals with ASD had a significantly less well-defined tissue contrast (i.e. GWPC) between gray and white matter at (and around) the gray–white matter boundary. The affected brain regions included the superior temporal gyrus (BA21), the dorsolateral frontal lobe (BA9), and the dorsal parietal lobe (BA7) where histological abnormalities in the transition from gray-to-white matter have also been reported (Avino and Hutsler 2010). The concordance between the regional pattern and direction of the GWPC differences in our sample with previous histological investigations in postmortem brain tissue supports the biological plausibility of our results. Thus, our findings agree with previous postmortem histological studies and indicate that tissue contrasts across the gray–white matter interface may serve as a potential in vivo proxy measure for atypical organization of the cortical sheet in ASD.

Prior postmortem studies reported abnormalities in the cortical microstructure of individuals with ASD. For example, the boundary between cortical layer VI and underlying white matter has been shown to be significantly less well defined due to increased dispersion of neuronal cells across this interface (Avino and Hutsler 2010). It has been suggested that this may be caused by the presence of supernumerary neurons beneath the cortical plate that arise from disrupted migratory processes or improper resolution of the cortical subplate (Chun and Shatz 1989; Kemper 2010; Hutsler and Avino 2015). The cortical subplate is a transient neurodevelopmental zone that is instrumental in establishing early proper cortical connectivity. Specifically, subplate neurons pioneer the corticothalamic axon pathway, serve as a “signpost” for cortical afferents, drive endogenous oscillatory activity in the cortex, and act as a transient synaptic hub for thalamocortical axons before they directly innervate the cortical plate (Shatz and Luskin, 1986; Ghosh et al., 1990; McConnell et al. 1994; Luhmann et al., 2009). The maximal volume of the subplate is reached around 30 gestational weeks in the human coinciding with the growth of long-range cortico–cortico projections (Vasung et al. 2016). After their early neurodevelopmental role is complete, a large number of these subplate neurons undergo apoptosis. However, a small percentage of these neurons persist and retain their connections with the overlying cortical plate acting as modulators of cortical afferents (Chun and Shatz 1989; Dupont et al. 2006; Kostović et al. 2011).

Therefore, perturbations to early subplate development may disrupt the establishment of structural and functional brain connectivity, which is abnormal in individuals with ASD (Belmonte et al. 2004; Just et al. 2004; Courchesne and Pierce 2005; Balardin et al. 2015). In addition, the abnormal persistence of these neurons after the large wave of programmed cell death could cause disruptions to cortical communication through their modulatory role of the overlying cortex. In this way, the abnormal persistence of subplate neurons into adulthood has been demonstrated in schizophrenia and seizure disorder and is hypothesized to contribute to the pathophysiology of these conditions (Eastwood and Harrison, 2003, 2005; Andres et al. 2005; Hildebrandt et al., 2005; Kostović et al. 2011; Yang et al., 2011). Furthermore, a recent genetic study reported a set of subplate-specific genes that are associated with ASD (Hoerder-Suabedissen et al. 2013). Thus, there is converging evidence to suggest that neurons of the cortical subplate contribute to the aberrant neuropathology of ASD and that atypical laminar organization, particularly around the gray–white matter boundary, may be a defining characteristic of the condition. However, this has never previously been examined in vivo.

Thus, in this in vivo study, we sought to examine differences in cortical lamination and gray–white matter boundary integrity in ASD. To achieve this, we measured contrasts between gray and white matter tissue intensities (GWPC; Salat 2009). These MRI measures were taken at the interface of gray and white matter and across cortical layers at 6 different depths into the cortical sheet from the gray–white matter boundary (i.e. white matter surface). In our ASD cases, many regions with reduced GWPC also showed significantly increased GMI but no differences in WMI as compared with TD controls. This suggests (in agreement with prior in vivo work by our group; Ecker et al. 2016) that ASD may be primarily associated with disruptions to cortical gray matter as opposed to white matter. This increased GMI in ASD may result from atypical myelination (Sowell et al. 2004) and/or atypical cytoarchitectural organization such as greater numbers of more densely packed cortical minicolumns (Casanova et al. 2006) and reductions in gray level amplitude in these structures (Casanova et al. 2002).

The regional specificity of our findings of decreased tissue contrast may be related to the differential expansion of the subplate between cortical areas. Evolutionarily, the size and complexity of the subplate is most prominent in humans as it accommodates the increased connectivity with cortical and subcortical areas relative to non-human primates and rodents (Kostovic and Rakic, 1990; Judas et al., 2013). Within humans, the subplate zone is larger in cortical association areas as a consequence of the increased number of axons invading these regions. These incoming axons displace subplate neurons deeper into the white matter, which occurs to a greater degree in these association areas (Duque et al. 2016). Atypicalities at the gray–white matter interface may therefore impact on MRI intensity values, and may explain the regional specificity observed in our pattern of results. Moreover, the regional pattern of GWPC seems to be linked to the functional deficits that are characteristic for ASD. For example, we observed deficits in GWPC in several regions mediating social processing and wider socio-cognitive functioning, including the insula, fusiform gyrus, cingulate cortex, middle temporal gyrus, superior temporal sulcus, and prefrontal cortical regions (see Just et al. 2012 for review). Thus, while future studies are required to establish the functional relevance of our results directly, it is likely that atypical GWPC contributes to the cluster of clinical symptoms typically observed in ASD.

Findings from this and other studies detailing poor delineation of the gray–white boundary in ASD may be taken by some to call into question the accuracy of in vivo MRI measures such as CT that rely on the placement of a discrete boundary between gray and white matter. However, the spatially distributed patterns of group-differences in CT we detected did not significantly overlap with the pattern of differences in GWPC (see Supplementary Fig. 1). Also, including individual's global mean CT as a covariate did not significantly alter the pattern of differences in GWPC. Therefore, while we were able to detect subtle differences in tissue contrast in ASD, at the level of spatial resolution neuroimaging techniques currently offer, these do not appear to be large enough to significantly affect estimates of CT within our sample of adults with ASD. This finding is also in agreement with a recent twin study showing that while both GWPC and CT are highly heritable, they have little shared genetic variance (Panizzon et al. 2012). Taken together, these findings suggest that GWPC characterizes additional cortical structural properties that are distinct to CT. Nevertheless, inter-individual differences in the ability to delineate the gray–white matter boundary should be considered in the future when interpreting neuroanatomical features that are based on clearly delineating gray and white matter.

Our study is not without limitations. For instance, we examined neuroanatomical differences associated with ASD in adulthood. This, and the cross-sectional nature of our study, inherently limits our ability to draw conclusions on the etiological and neurodevelopmental basis of the atypical neural structure we observed. However, within our sample, all but 4 females with ASD met ADI-R criteria for childhood autism. It is therefore likely that the observed pattern of neuroanatomical differences in GWPC may have evolved as a consequence of meeting ASD criteria during early childhood and is therefore causally related to the condition. Further longitudinal studies will, however, be required to disentangle GWPC differences associated with primary neuropathology from atypical neurodevelopmental trajectories or secondary compensatory mechanisms. Recent work has quantified the volume of transient neurodevelopmental zones in the postmortem human fetal brain using MRI as they relate to major neurogenic events (Vasung et al. 2016). Such information provides a reference for studying early prenatal deviations from TD brain growth and could be used in the future to inform in vivo imaging. We are further limited by the current resolution of structural MRI images (1 mm isotropic voxels). At this resolution, it is not possible for us to distinguish between different aspects of cortical cytoarchitecture or accurately delineate particular layers of the cortical sheet as defined by histological staining. Rather, our sampling approach was based on the geometric criteria of projection fraction percentages into the cortical sheet from the white matter surface (Salat et al. 2009). Furthermore, additional research will be required to elucidate the functional relationship between deficits in GWPC and autistic symptoms and traits.

Taken together, our findings suggest that measures of GWPC sampled across cortical layers may serve as an in vivo proxy measure for irregular microstructural organization of the cortex in ASD (and other disorders). Such novel in vivo measures that are indicative of atypical cortical organization might in the future be used to stratify the condition, and/or to examine the neuropathology of ASD in particular genetic subgroups known to be linked to specific neurodevelopmental deficits.

## Supplementary Material

Supplementary material are available at *Cerebral Cortex* online.

## Funding

The study represents independent research partly funded by the National Institute for Health Research (NIHR) Biomedical Research Centre at South London and Maudsley NHS Foundation Trust and King's College London. The views expressed are those of the authors and not necessarily those of the NHS, the NIHR or the Department of Health. This work was also supported by funding from the Medical Research Council UK (grant number G0400061); the Innovative Medicines Initiative Joint Undertaking (grant number 115300), which includes financial contributions from the EU Seventh Framework Programme (FP7/2007-2013) from the European Federation of Pharmaceutical Industries and Associations companies in kind; and from Autism Speaks.

## Supplementary Material

Supplementary Data

Supplementary Data

Supplementary Data

Supplementary Data

Supplementary Data
