# Supplementary figures and images for: In Vivo Evidence of Reduced Integrity of the Gray–White Matter Boundary in Autism Spectrum Disorder
Source: Cereb Cortex. 2017 Jan 5;27(2):877–87. doi: 10.1093/cercor/bhw404 (PMC6093436; doi:10.1093/cercor/bhw404)

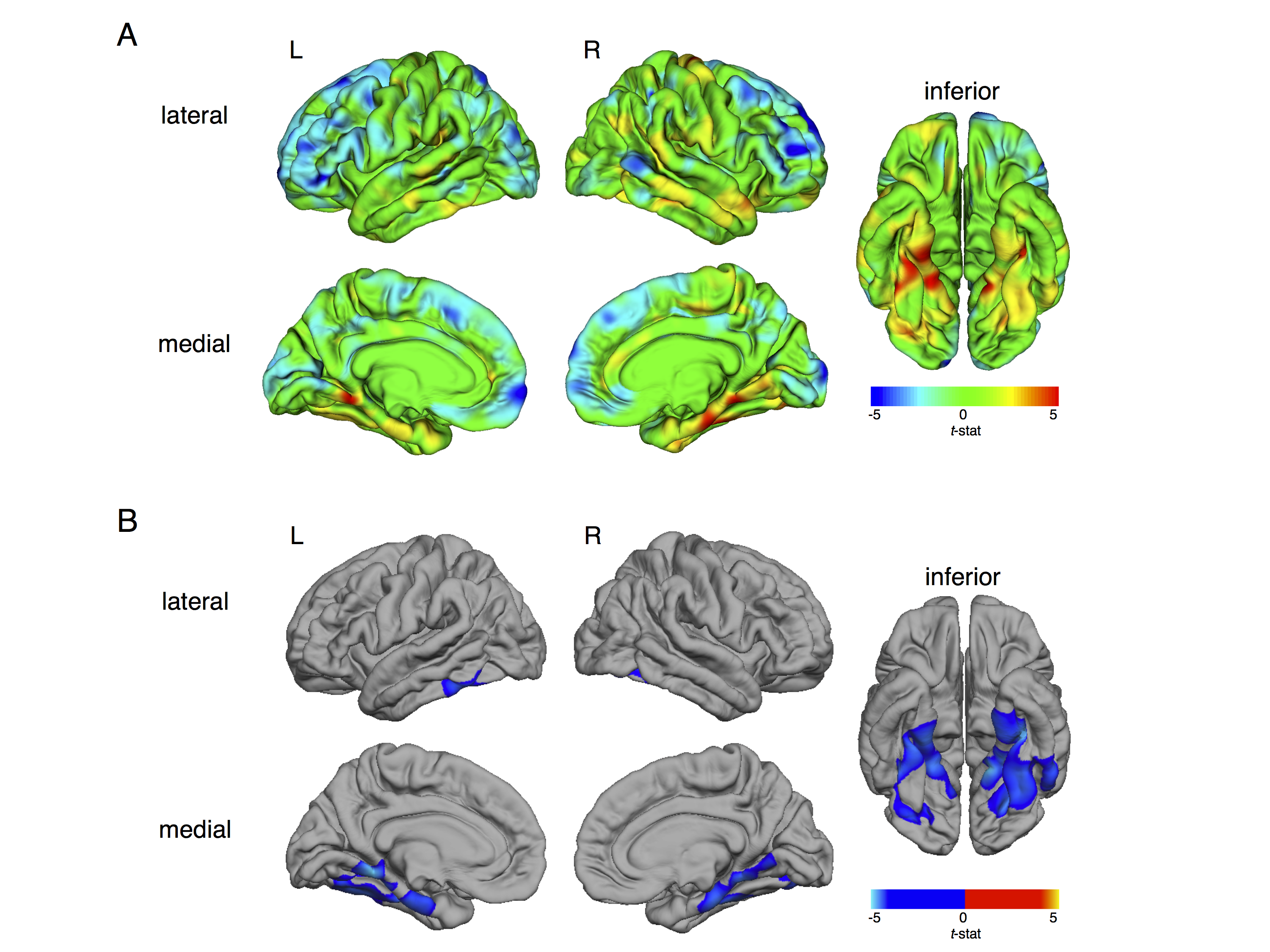

Supplement: Supplementary Data [file supfig1.png]

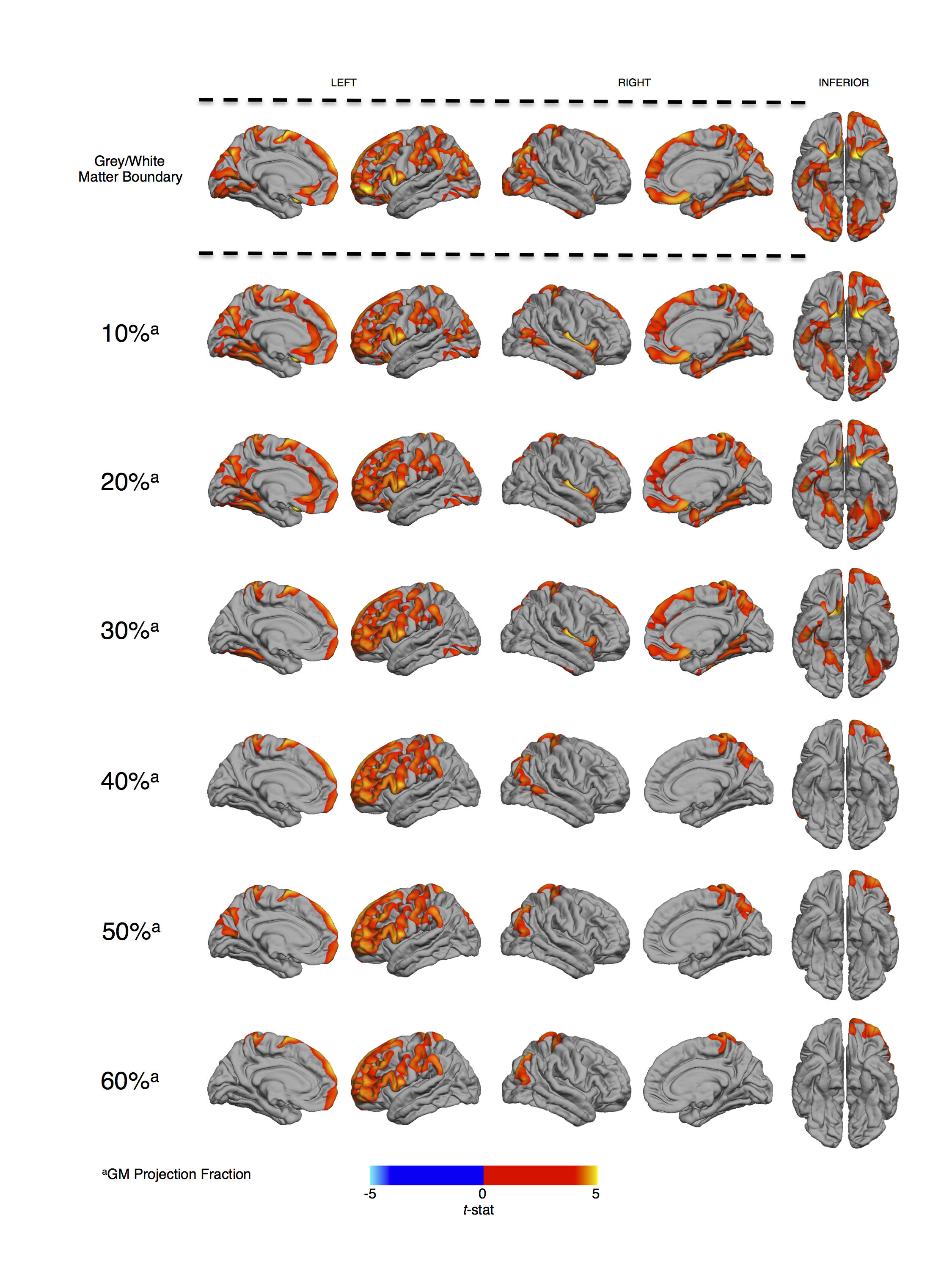

Supplement: Supplementary Data [file supfig2.png]

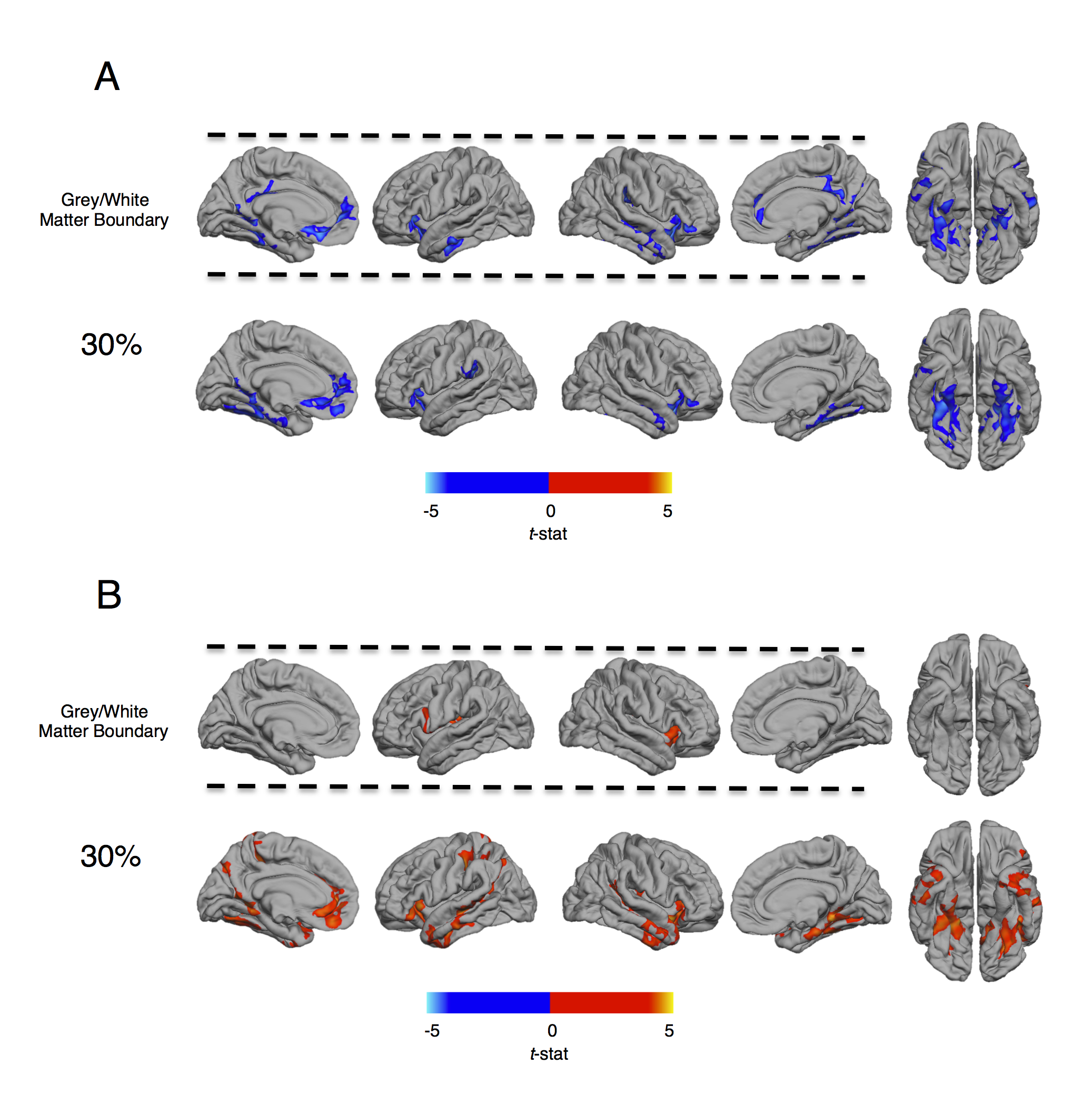

Supplement: Supplementary Data [file supfig3.png]
