# Supplementary material for: In Vivo Evidence of Reduced Integrity of the Gray–White Matter Boundary in Autism Spectrum Disorder
Source: Cereb Cortex. 2017 Jan 5;27(2):877–87. doi: 10.1093/cercor/bhw404 (PMC6093436; doi:10.1093/cercor/bhw404)
Supplement: Supplementary Data [file supmaterials_supfigcaptions.docx]

**Supplementary Materials**

*Surface deformation procedure to place the grey-white matter boundary (i.e. white matter surface)*

Within this study we refer to the white matter surface (i.e. the surface that defines the transition from grey to white matter) as the grey-white matter boundary. The surface deformation procedure that places the white matter surface has previously been described by Dale et al. (1999) and is detailed bellow.

First white matter voxels are labeled through a segmentation procedure. Contiguous white matter voxels are identified through a connected components algorithm resulting in a filled white matter labeled volume. This volume is then tessellated using two triangles to define each voxel composing the surface of the white matter volume. Deformation of this “jagged” white matter tessellation to the grey-white matter boundary is accomplished by a minimization of an energy functional. The first two terms of this energy functional act to smooth the surface and regularize the tessellation by introducing a spring like property to the surface. This spring property is decomposed into two terms given as,

$$J_{n}=\frac{1}{2V}\left( \sum_{i=1}^{V} \sum_{j\in N_{1}i} (\mathbf{n}(i)\cdot(\mathbf{x}_{i}-\mathbf{x}_{j}))^{2} \right)$$

$$J_{t}=\frac{1}{2V}\left( \sum_{i=j}^{V} \sum_{j\in N_{1}i} (\mathbf{e}_{0}\left( i \right)\cdot\left( \mathbf{x}_{i}-\mathbf{x}_{j} \right))^{2}+(\mathbf{e}_{1}\left( i \right)\cdot\left( \mathbf{x}_{i}-\mathbf{x}_{j} \right))^{2} \right)$$

where N_1_(i) denotes the set of nearest neighbors of the i^th^ vertex, V is the total number of vertices in the tessellation, **n**(i) is the unit normal vector to the surface at the i^th^ vertex, [**e**_0_(i),**e**_1_(i)] is an orthonormal basis for the tangent plane at the i^th^ retex, and **x**_k_ regers to the (x,y,z) position of the k^th^ vertex in the tessellation. The term J_i_ results in the redistribution of vertices to regions where they are needed, encouraging a uniform spacing of vertices without requiring prohibitive numbers of elements. The term J_n_ imposes a smoothness constraint on the surface deformation by penalizing nodes that distance themselves from the direction normal to surface from its neighboring nodes. The third term of the energy functional is based on intensity values. The volume intensity at position x_i_ can be written as I(x_i_) and this term given as,

$$J_{I}=\frac{1}{2V}\left( \sum_{i=1}^{V} (T\left( i \right)-I(x_{i}))^{2} \right)$$

where T(i) is the mean white matter value of border voxels within a 5mm neighborhood of each vertex, within the segmented white matter volume. The value of I(x) is computed on a subvoxel basis using trilinear interpolation. The placement of the grey-white matter boundary is achieved by minimizing an energy function that is a weighted sum of the three terms presented above,

$$J=J_{t}+ \lambda_{n}J_{n}+ \lambda_{I}J_{I}$$

where the coefficients $\lambda_{n}$ and $\lambda_{I}$ specify the strength of the smoothness and regularization constraints in relation to the intensity term. The gradient of this functional defines the movement of the surface tessellation such as the movement of the k^th^ vertex is given by the negative of the directional derivative with respect to **x**_k,,_

$$-\frac{\partial J}{\partial\mathbf{x}_{k}}=\lambda_{I}\left( T\left( k \right)-I\left( \mathbf{X}_{k} \right) \right)\nabla I\left( \mathbf{x}_{k} \right)+\sum_{j\in N_{1}(k)} (\lambda_{n}\left( \mathbf{n}\left( k \right)\cdot\mathbf{x}_{j} \right)+\mathbf{e}_{0}(k)\cdot\mathbf{x}_{j}+\mathbf{e}_{1}(k)\cdot\mathbf{x}_{j})$$

where the volume gradient $\nabla I\left( \mathbf{x}_{k} \right)$ is computed using a Gaussian blurred $(\sigma=1)$ version of the MRI volume.

These automated methods for determining the grey-white matter boundary have been previously validated using scans of postmortem brains and have found FreeSurfer based measures of cortical thickness to be on average only 0.077mm different than manual measures performed on dissected tissue samples (Rosas et al. 2002). Within group systematic errors in the placement of the grey-white matter boundary using these methods would result in whole brain differences in cortical thickness that are not observed in our study. These findings thus indicate a high degree of accuracy for FreeSurfer in placing the white matter surface (i.e. the grey-white matter boundary).

**Supplementary Figure Captions**

**Supplementary Figure 1, Regions of decreased cortical thickness (CT) in autism spectrum disorder (ASD):** A.) Between group differences in CT (uncorrected). B.) Individuals with ASD showed significantly decreased CT (RFT *p*<0.5) bilaterally in the parahippocampal, fusiform, and lingual gyri (highlighted in blue). See supplementary Table 1 for statistical details of these clusters.

**Supplementary Figure 2, Sex differences in grey-white matter signal intensity percent contrast (GWPC):** Regardless of diagnosis males showed significantly greater GWPC (RFT *p*<0.5) compared to females across all grey matter sampling depths (a). These increases are highlighted in red and include predominantly fronto-parietal regions of the left hemisphere, and bilateral inferior temporal regions (see supplementary Table 2 for statistical details of these clusters).

**Supplementary Figure 3, Regional differences in grey-white matter signal intensity percent contrast (GWPC) and grey matter intensities (GMI) in Autism Spectrum Disorder (ASD) (5mm FWHM smoothing kernel):** Between group differences in (A) GWPC and (B) GMI intensities are shown when GMI was sampled at the grey-white matter boundary (i.e. white matter surface, projection fraction 10%) and a projection fraction of 30% into the cortical sheet. Individuals with ASD showed (A) significantly decreased GWPC (RFT *p*<0.5), indicating less definition between grey and white matter, in several regions highlighted in blue. In several of these regions (B) increases in GMI, highlighted in red were also observed. These results using a 5mm FWHM smoothing kernel were largely similar to those using a 10mm FWHM smoothing kernel (Figures 2 and 3, Table 2). For statistical details of these clusters see supplementary Table 3.
