# Supplementary material for: In Vivo Evidence of Reduced Integrity of the Gray–White Matter Boundary in Autism Spectrum Disorder
Source: Cereb Cortex. 2017 Jan 5;27(2):877–87. doi: 10.1093/cercor/bhw404 (PMC6093436; doi:10.1093/cercor/bhw404)
Supplement: Supplementary Data [file supplementarytables.docx]

| **Supplementary Table 1** | | | | | | | | | | | | |
| --- | --- | --- | --- | --- | --- | --- | --- | --- | --- | --- | --- | --- |
|  |  |  |  |  |  | | Talairach | | | |  |  |
| Cluster | Region Labels | | Hemisphere | BA(*t*_max_) | | No vertices | x | y | | z | *t*_max_ | *p*_cluster_ |
|  | | | | | | | | | | | | |
| 1 | parahippocampal gyrus, entorhinal cortex, fusiform gyrus, inferior temporal gyrus, lingual gyrus | | L | 19 | | 6191 | -17 | | -48 | -3 | -4.46 | 4.89 x 10-6 |
| 2 | parahippocampal gyrus, fusiform gyrus, lingual gyrus | | R | 36 | | 3728 | 21 | | -42 | -5 | -3.35 | 1.73 x 10-4 |

**Supplementary Table 1, Clusters of Decreased Cortical Thickness in Autism Spectrum Disorder (ASD):** Broadmann area (BA), left (L), right (R), *Vertices* indicates the number of vertices within the cluster, *t_max_* represents the maximum *t*-statistic within the cluster located at the x y z Talairach coordinates listed, *p_cluster_* is the cluster corrected *p* value.

| **Supplementary Table 2** | | | | | | | | | | |
| --- | --- | --- | --- | --- | --- | --- | --- | --- | --- | --- |
|  |  |  |  |  |  | Talairach | | |  |  |
|  | Cluster | Region Labels | Hemisphere | BA*(t*_max_) | No. vertices | x | y | z | *t*_max_ | *p*_cluster_ |
|  |  |  |  |  |  |  |  |  |  |  |
|  | 1 | **precentral gyrus**, frontal pole, pars opercularis, pars orbitalis, pars triangularis, rostral middle frontal gyrus, superior frontal gyrus | L | 44 | 11867 | -49 | 10 | 6 | 5.35 | 4.38 x 10^-6^ |
|  | 2 | **inferior parietal cortex**, lateral occipital cortex, lingual gyrus, middle temporal gyrus, superior parietal cortex | R | 7 | 10199 | 39 | -63 | 44 | 4.82 | 4.38 x 10^-6^ |
|  | 3 | **middle temporal gyrus**, inferior parietal cortex, lateral occipital cortex, postcentral gyrus, superior parietal cortex, supramarginal gyrus | L | 19 | 9991 | -38 | -78 | 25 | 4.49 | 4.38 x 10^-6^ |
|  | 4 | **precuneus**, inferior temporal gyrus, isthmus-cingulate cortex, lateral occipital cortex, lingual gyrus, pericalcarine cortex, superior parietal cortex | L | 7 | 9359 | -6 | -67 | 41 | 4.53 | 4.38 x 10^-6^ |
|  | 5 | **postcentral gyrus**, paracentral lobule, precentral gyrus, precuneus cortex, superior parietal cortex | R | 3 | 7611 | 36 | -30 | 61 | 4.54 | 4.38 x 10^-6^ |
|  | 6 | **parahippocampal gyrus**, entorhinal cortex, fusiform gyrus, inferior temporal gyrus | R | 36 | 4001 | 34 | -27 | -15 | 3.42 | 1.16 x 10^-3^ |
|  | 7 | **lateral orbital frontal cortex**, medial orbital frontal cortex, rostral anterior cingulate cortex | L | 47 | 3563 | -24 | 12 | -16 | 5.71 | 1.14 x 10^-3^ |
|  | 8 | **superior frontal gyrus** | R | 6 | 3139 | 9 | 20 | 54 | 4.59 | 8.45 x 10^-4^ |
|  | 9 | **lateral orbital frontal cortex**, medial orbital frontal cortex | R | 47 | 2824 | 26 | 10 | -14 | 5.29 | 1.06 x 10^-2^ |
|  | 10 | **middle frontal gyrus**, precentral gyrus | L | 9 | 2246 | -37 | 32 | 25 | 4.31 | 1.81 x 10^-3^ |
|  | 11 | **paracentral lobule**, precentral gyrus, superior parietal cortex | L | 6 | 1988 | -9 | -25 | 67 | 3.79 | 2.20 x 10^-2^ |

**Supplementary Table 2, Clusters of Increased Grey-White Matter Signal Intensity Percent Contrast (GWPC) in Males:** Broadmann area (BA), left (L), right (R), *Vertices* indicates the number of vertices within the cluster, *t_max_* represents the maximum *t*-statistic within the cluster located at the x y z Talairach coordinates listed, *p_cluster_* is the cluster corrected *p* value.

| **Supplementary Table 3** | | | | | | | | | | |
| --- | --- | --- | --- | --- | --- | --- | --- | --- | --- | --- |
|  |  |  |  |  |  | Talairach | | |  |  |
| Measure | Cluster | Region Labels | Hemisphere | BA(*t*max) | Vertices | x | y | z | *t*max | *p*cluster |
| Grey-White Matter  Percent Contrast |  |  |  |  |  |  |  |  |  |  |
|  | 1 | fusiform gyrus, lingual gyrus, parahippocampal gyrus | L | 36 | 4027 | -27 | -39 | -7 | -3.66 | 1.33 x 10-5 |
|  | 2 | parahippocampal gyrus, fusiform gyrus, lingual gyrus | R | 20 | 3447 | 33 | -35 | -18 | -3.8 | 1.33 x 10-5 |
|  | 3 | medial orbital frontal cortex, rostral anterior cingulate cortex, superior frontal gyrus | L | 10 | 2342 | -10 | 39 | -4 | -3.68 | 1.45 x 10-5 |
|  | 4 | insula, lateral orbital frontal cortex | R | 47 | 1671 | 26 | 18 | -14 | -4.02 | 1.76 x 10-4 |
|  | 5 | posterior-cingulate cortex, isthmus-cingulate cortex, lingual gyrus, precuneus cortex | L | 30 | 1348 | -19 | -53 | 9 | -3.72 | 3.52 x 10-4 |
|  | 6 | insula, lateral orbital frontal cortex | L | 13 | 1267 | -30 | 19 | -2 | -3.58 | 1.05 x 10-3 |
|  | 7 | middle temporal gyrus, superior temporal gyrus | R | 21 | 1068 | 55 | -13 | -17 | -3.74 | 1.01 x 10-2 |
|  | 8 | insula | L | 13 | 1132 | -38 | -4 | 16 | -2.97 | 4.43 x 10-2 |
|  | 9 | supramarginal gyrus | R | 13 | 926 | -45 | -32 | 23 | -4.13 | 1.24 x 10-2 |
| Grey Matter Signal Intensity |  |  |  |  |  |  |  |  |  |  |
|  | 1 | insula, lateral orbital frontal cortex, superior temporal gyrus | R | 38 | 4370 | 37 | 0 | -12 | 4.01 | 7.28 x 10-6 |
|  | 2 | banks superior temporal sulcus, inferior parietal cortex, middle and superior temporal gyrus | L | 21 | 4021 | -50 | -26 | -2 | 3.64 | 7.89 x 10-6 |
|  | 3 | banks superior temporal sulcus, inferior parietal cortex, inferior, middle, and superior temporal gyri | R | 41 | 3735 | 46 | -36 | 7 | 2.95 | 1.05 x 10-5 |
|  | 4 | fusiform gyrus, lingual gyrus, parahippocampal gyrus | R | 30 | 3522 | 18 | -38 | -6 | 3.90 | 7.40 x 10-6 |
|  | 5 | isthmus-cingulate cortex, precuneus cortex | L | 29 | 2694 | -14 | -49 | 7 | 3.38 | 3.39 x 10-5 |
|  | 6 | fusiform gyrus, inferior temporal gyrus, lingual gyrus | L | 19 | 2410 | -29 | -56 | -3 | 3.56 | 2.22 x 10-4 |
|  | 7 | medial orbital frontal cortex, rostral anterior cingulate cortex, superior frontal gyrus | L | 32 | 2165 | -11 | 42 | 2 | 3.40 | 2.96 x 10-4 |
|  | 8 | postcentral gyrus, superior parietal cortex | L | 2 | 1868 | -46 | -23 | 43 | 3.56 | 1.66 x 10-2 |
|  | 9 | insula, lateral orbital frontal cortex | L | 13 | 1654 | -30 | 18 | -2 | 3.79 | 4.09 x 10-3 |
|  | 10 | paracentral lobule, superior parietal cortex, | L | 5 | 1458 | -16 | -35 | 50 | 3.11 | 4.85 x 10-2 |
|  | 11 | superior temporal gyrus | L | 21 | 1228 | -45 | -9 | -13 | 2.78 | 2.73 x 10-2 |
|  | 12 | inferior temporal gyrus | L | 20 | 1085 | -48 | -10 | -23 | 3.47 | 3.34 x 10-2 |

**Supplementary Table 3, Significant Reductions in Grey-White Matter Signal Intensity Percent Contrast (GWPC) and Increases in Grey Matter Intensity (GMI) in ASD (FWHM 5mm):** Broadmann area (BA), left (L), right (R), *Vertices* indicates the number of vertices within the cluster, *t_max_* represents the maximum *t*-statistic within the cluster located at the x y z Talairach coordinates listed, *p_cluster_* is the cluster corrected *p* value.
